# Long Noncoding RNA GATA3-AS1 Promotes Cell Proliferation and Metastasis in Hepatocellular Carcinoma by Suppression of PTEN, CDKN1A, and TP53

**DOI:** 10.1155/2019/1389653

**Published:** 2019-12-02

**Authors:** Xuee Luo, Ning Zhou, Le Wang, Qinghua Zeng, Hongying Tang

**Affiliations:** Laboratory of Hepatobiliary Molecular Oncology, Department of Hepatopancreatobiliary Surgery, Hunan Provincial People's Hospital, The First Affiliated Hospital of Hunan Normal University, Changsha 410011, Hunan Province, China

## Abstract

**Background:**

Long noncoding RNAs (lncRNAs) have been known to play important roles in the progression of various types of human cancer. LncRNA GATA3 antisense RNA 1, GATA3-AS1, has been reported to be associated with T-cell development and differentiation. However, the expression pattern and function of GATA3-AS1 in hepatocellular carcinoma (HCC) remain unknown.

**Methods:**

Real-time quantitative PCR (RT-qPCR) assay was conducted to detect GATA3-AS1 expression levels in 80 cases of pairs HCC tissues and matched normal tissues. Chi-squared (*χ*^2^) test was used to analyze the correlation between GATA3-AS1 expression and clinicopathologic variables. Survival curves were plotted using the Kaplan–Meier method and were compared via the log-rank test. The cell counting kit-8 (CCK-8) and wound scratch assays were applied to detect the effect of GATA3-AS1 knockdown and overexpression on cell growth and migration of HCC. RT-qPCR was performed for the detection of the phosphatase and tensin homolog (PTEN), cyclin-dependent kinase inhibitor 1A (CDKN1A), and tumor protein p53 (TP53) expression in HCC cells after GATA3-AS1 knockdown and overexpression.

**Results:**

GATA3-AS1 was significantly upregulated in HCC tissues compared with matched normal tissues. The high expression of GATA3-AS1 was significantly correlated with larger tumor size, advanced TNM stage, and more lymph node metastasis. High GATA3-AS1 expression was markedly correlated with shorter overall survival times of HCC patients. Furthermore, knockdown of GATA3-AS1 obviously inhibited Hep3B and HCCLM3 cell growth and migration, whereas overexpression of GATA3-AS1 had the opposite effects. In addition, GATA3-AS1 knockdown resulted in upregulated expression levels of tumor-suppressive genes, PTEN, CDKN1A, and TP53, in Hep3B and HCCLM3 cells, while restoration of GATA3-AS1 decreased PTEN, CDKN1A, and TP53 expression levels.

**Conclusion:**

Our data suggested that GATA3-AS1 promotes cell proliferation and metastasis of HCC by suppression of PTEN, CDKN1A, and TP53.

## 1. Introduction

Hepatocellular carcinoma (HCC) is one of the most common diagnosed malignancies and the second leading cause of tumor-related deaths worldwide [1]. According to the global cancer statistics, approximately 500,000 new cases and 600,000 cases of mortalities occur every year [2]. The long-term prognosis of HCC remains very poor, with a low 5-overall survival rate of 35% [3]. Surgical removal of the tumor and liver transplant are currently the most applicable options for HCC; however, tumor metastasis and recurrence are extremely common in postoperative HCC patients [4, 5]. At present, because of the poor understanding of pathological molecular mechanisms in HCC, the effective therapy for this cancer is very limited [6]. Therefore, it is essential to identify the mechanisms that underlie HCC metastasis for the development of novel sensitive and effective therapies.

Long noncoding RNAs (lncRNAs) are a class of noncoding RNAs which surpass 200 nucleotides in length [7]. According to the chromosome region to their adjacent coding genes, lncRNAs can be divided into five categories, including sense, antisense, bidirectional, intronic, and intergenic lncRNAs [8]. Accumulating data demonstrated that lncRNAs play vital roles in various biological processes, such as cell growth and differentiation, immune activation/inactivation, and transcriptional and posttranscriptional regulation [9]. Recent studies have identified that some of lncRNAs were closely participated in tumorigenesis and metastasis and can be used as the effective biomarkers for HCC [10, 11]. Therefore, more and more researchers are keen on the eyes of lncRNAs in the progression of HCC.

In recent years, antisense lncRNAs have been identified to regulate cell proliferation, migration, and invasion in cancer [12]. For example, lncRNA antisense transcript of coding gene PCNA, PCNA-AS1, is shown to increase HCC cell growth [13]. The lncRNA MDC1 antisense RNA 1 (MDC1-AS1) associates with the development of bladder cancer [14]. The lncRNA AFAP1 antisense RNA 1, AFAP1-AS1, plays an oncogenic role in promoting cell migration in non-small cell lung cancer [15]. Besides, lncRNA DLX6 antisense RNA 1 (DLX6-AS1) promotes malignant phenotypes of gastric cancer cells [16]. The GATA3 antisense RNA 1 (GATA3-AS1) is a new antisense lncRNA, which is associated with T-cell development and differentiation [17]. It was later confirmed that GATA3-AS1 regulates GATA3 transcription in T-helper 2 cells [18]. However, the expression levels and function of GATA3-AS1 in HCC remain unknown. In this study, we firstly determined GATA3-AS1 expression in HCC tissues and cells. Then, we performed *in vitro* loss- and gain-of-function experiments for GATA3-AS1 in the progression of HCC. This study highlighted the oncogenic role of GATA3-AS1 in regulating HCC cell proliferation and metastasis.

## 2. Materials and Methods

### 2.1. HCC Specimens

Human HCC tissue and matched normal tissues were randomly collected from Department of Hepatopancreatobiliary Surgery, Hunan Provincial People's Hospital (Changsha, China) between June 2006 and December 2015. A total of 80 cases of HCC were enrolled in the study. All tissues were instantly frozen in liquid nitrogen after operation until use. This research was approved by the Institutional Review Board of Hunan Provincial People's Hospital (no. 2017064). The written informed consent was obtained from all patients or their relatives based on the Declaration of Helsinki. The HCC stage was classified according to the modified tumor-node-metastasis (TNM) cancer staging system published by the International Union Against Cancer (UICC, 2009) [19]. The clinicopathological information is summarized in Table 1.

### 2.2. Cell Culture

Two human HCC cells (Hep3B and HCCLM3) and the normal liver cell line (HL-7702) were obtained from the Shanghai Institute of Biochemistry and Cell Biology, Chinese Academy of Sciences (Shanghai, China). The Hep3, HCCLM3, and HL-7702 cells were maintained in the RPMI 1640 medium (Invitrogen, Carlsbad, CA, USA) plus 10% FBS (Invitrogen, Carlsbad, CA, USA) with 100 U/ml penicillin and 100 *μ*g/ml streptomycin at 37°C with 5% CO_2_.

### 2.3. Transfection

To knockdown an endogenous GATA3-AS1 expression, GATA3-AS1 small interfering RNA (siRNA) (sequence: 5′-UCUCCGCGCGUCAAUCGA-3′) and control siRNA (sequence: 5′-CUACACCGUAUUCUACUACUA-3′) were obtained from Genechem Co., Ltd. (Shanghai, China). To overexpress GATA3-AS1, pcDNA3.1 + GATA3-AS1 recombinant plasmid was constructed by inserting an EcoRI-Xhol fragment containing the GATA3-AS1 transcript into the same sites in pcDNA3.1 + vector. The pcDNA3.1 + vector (empty vector) was used as a negative control for pcDNA3.1 + GATA3-AS1 plasmid. The total GATA3-AS1 transcript was amplified from HCCLM3 cells by RT-PCR using the specific primers: 5′-GCTGCAGCC-GCTGGCCCGAAAATGC-3' (sense) and 5′-TTCTAAAG-GTGGGGGTTGCCCTTCT-3' (antisense). The primers were bought from Life Technologies (Invitrogen, Carlsbad, CA, USA), and the sequences of recombinant plasmid were confirmed in Life Technologies (Invitrogen) by Sanger DNA sequencing. For cell transfection, Hep3B and HCCLM3 cells were transfected with 200 pmol GATA3-AS1 siRNA/control siRNA or 4 *μ*g pcDNA3.1 + GATA3-AS1 vector/empty vector by using Lipofectamine 2000 transfection reagent (Invitrogen) according to the instructions. The cells were collected, and transfection efficiency was examined using real-time quantitative PCR (RT-qPCR) assay after 48 h transfection.

### 2.4. RT-qPCR

Total RNA was extracted from HCC tissues and cells using TRIzol reagent (Invitrogen, Carlsbad, CA, USA) according to the manufacturer's instructions. The eligible RNA (OD 260/280 at 1.8–2.0) was converted to cDNA using the M-MLV Reverse Transcriptase kit (Toyobo, Osaka, Japan). After that, qPCR was carried out using a SYBR_Premix ExTaq II kit (Toyobo) on Applied Biosystems 7500 Real-Time PCR system (Applied Biosystems, Foster City, CA) to quantify the relative expression of target genes. The specific primers used for the amplification were shown in Table 2. ACTB (actin beta) was used as the internal control. The average of three independent data for each gene was calculated using 2^−ΔΔ*Cq*^ method [20].

### 2.5. Cell Proliferation Assay

The proliferation ability of HCC cells was detected using the cell counting kit-8 (CCK-8) assay (Dojindo Laboratories, Kumamoto, Japan). About 6000 cells of Hep3B and HCCLM3 were plated in 96-well plates per well and cultured in the RPMI 1640 medium for 24 h. Then, cells were treated with 50 pmol GATA3-AS1 siRNA/control siRNA or 0.5 *μ*g pcDNA3.1 + GATA3-AS1 vector/empty vector using lipofectamine 2000 transfection reagent, according to the manufacturer's instructions. 10 *μ*l CCK-8 solution/well was added, and cell proliferation ability was determined 0, 24, 48, and 72 h after transfection. The numbers of proliferative cells per well were measured at the absorbance (480 nm) by using a microplate reader (Bio-Rad, Hercules, CA, USA) at the indicated time points.

### 2.6. Wound Scratch Assay

Wound scratch assay was used to evaluate the metastatic ability of Hep3B and HCCLM3 cell lines *in vitro*. Approximately 8 × 10^6^ cells were seeded into per 6-well plates and transfected with 200 pmol GATA3-AS1 siRNA/control siRNA or 4 *μ*g pcDNA3.1 + GATA3-AS1 vector/empty vector. After 6 h transfection, the cell monolayer was scraped using a yellow pipette tip. The beginning gap length at 0 h and the residual gap length at 12 h were detected using the Image Analysis and Detection System (MIAS) (Leica Microsystems GmbH, Wetzlar, Germany). The experiments were conducted in triplicate and repeated at least three times and then analyzed by at least two observers with a double-blind manner.

### 2.7. Statistical Analysis

Data were presented as mean ± SD (standard deviation) of three independent experiments. The statistical analyses were performed using SPSS 17.0 (SPSS Inc., Chicago, IL, USA). The expression differences between HCC tissues and matched normal tissues were analyzed using paired student's *t*-test. Chi-squared (*χ*^2^) test was used for correlation analysis. Survival curves were plotted using the Kaplan–Meier method and were compared via the log-rank test. CCK-8 assay was analyzed using one way-ANOVA following Bonferroni's post hoc test. The value of *P* < 0.05 was considered to be a statistically significant difference.

## 3. Results

### 3.1. The GATA3-AS1 Expression Levels Are Markedly Upregulated in HCC Tissues and Cell Lines

**Figure 1:**
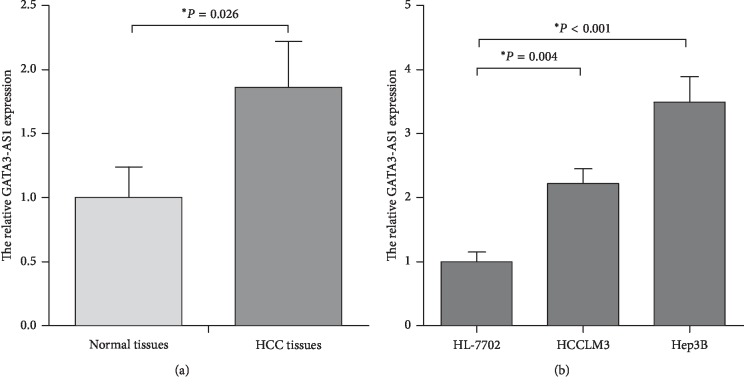
The lncRNA GATA3-AS1 expression levels are markedly upregulated in HCC tissues and cell lines. (a) GATA3-AS1 expression levels were examined in 80 cases of pairs HCC tissues and matched normal tissues using RT-qPCR analysis. The 2^−ΔΔCq^ method was used to analyze the results, and ACTB was used as the internal control. (b) GATA3-AS1 expression levels were detected by RT-qPCR assay in the two human HCC cell lines (Hep3B and HCCLM3) and the normal liver cell line (HL-7702). LncRNA: long noncoding RNA, GATA3-AS1 :GATA3 antisense RNA 1, HCC: hepatocellular carcinoma, RT-qPCR: real-time quantitative PCR, and ACTB: actin beta. Data were expressed as the mean ± SD (*n* = 3); ^*∗*^*P* < 0.05.

In order to identify the clinical significance of GATA3-AS1 in HCC patients, we firstly determined the GATA3-AS1 expression in HCC specimens. As shown in Figure 1(a), the GATA3-AS1 expression was significantly upregulated in HCC tissues compared with matched normal tissues (*P* < 0.05). We then analyzed the expression levels of GATA3-AS1 in two human HCC cells (Hep3B and HCCLM3) and the normal liver cell line (HL-7702). Consistent with the data of HCC specimens, GATA3-AS1 expression was obviously increased in the Hep3B and HCCLM3 cells compared with the HL-7702 cells (Figure 1(b), *P* < 0.05). The results indicated that upregulation of GATA3-AS1 may associate with the progression of HCC.

### 3.2. Upregulation of GATA3-AS1 Is Associated with the Aggressive Phenotypes and Poor Prognosis in HCC Patients

**Figure 2:**
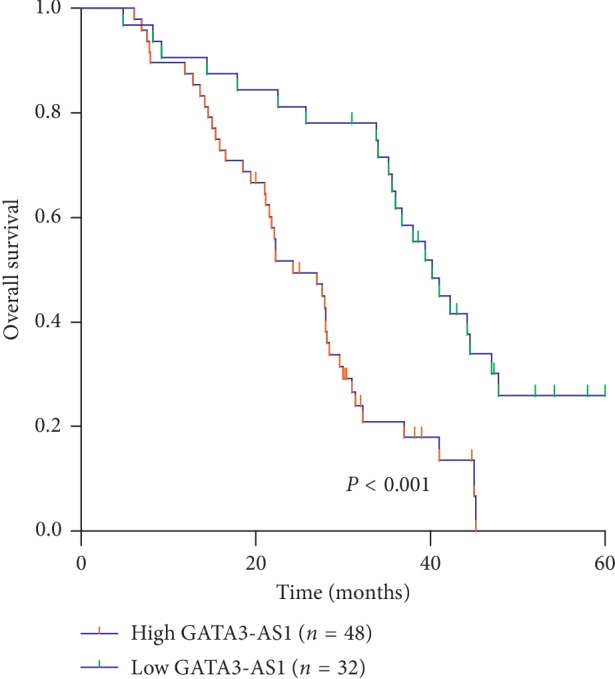
The association between GATA3-AS1 expression and prognosis of patients with HCC. Kaplan–Meier survival analysis and log-rank test indicated that high GATA3-AS1 expression was markedly correlated with shorter overall survival times of HCC patients.

The 80 pairs of HCC cases were divided into two groups based on the mean value of relative GATA3-AS1 expression levels, including low (*n* = 32) and high (*n* = 48) GATA3-AS1 expression groups. The *χ*^2^ test was used to analyze the correlation between GATA3-AS1 expression and clinicopathologic variables. As shown in Table 1, the high level of GATA3-AS1 expression was strongly correlated with larger tumor size (*P*=0.006), advanced TNM stage (*P*=0.003), and more lymph node metastasis (*P* < 0.001) but not correlated with patient's gender (*P*=0.506), age (*P*=0.168), differentiation (*P*=0.372), vascular invasion (*P*=0.126), and liver cirrhosis (*P*=0.920). Furthermore, Kaplan–Meier survival analysis showed a clear negative correlation between GATA3-AS1 expression and overall survival of HCC patients (Figure 2). The patients with high levels of GATA3-AS1 expression had a significantly shorter overall survival times compared with those with low GATA3-AS1 expression (*P* < 0.001). These results indicated that high expression of GATA3-AS1 is associated with the aggressive phenotypes and poor prognosis of HCC patients.

### 3.3. GATA3-AS1 Promotes Cell Proliferation in Hep3B and HCCLM3 Cells

**Figure 3:**
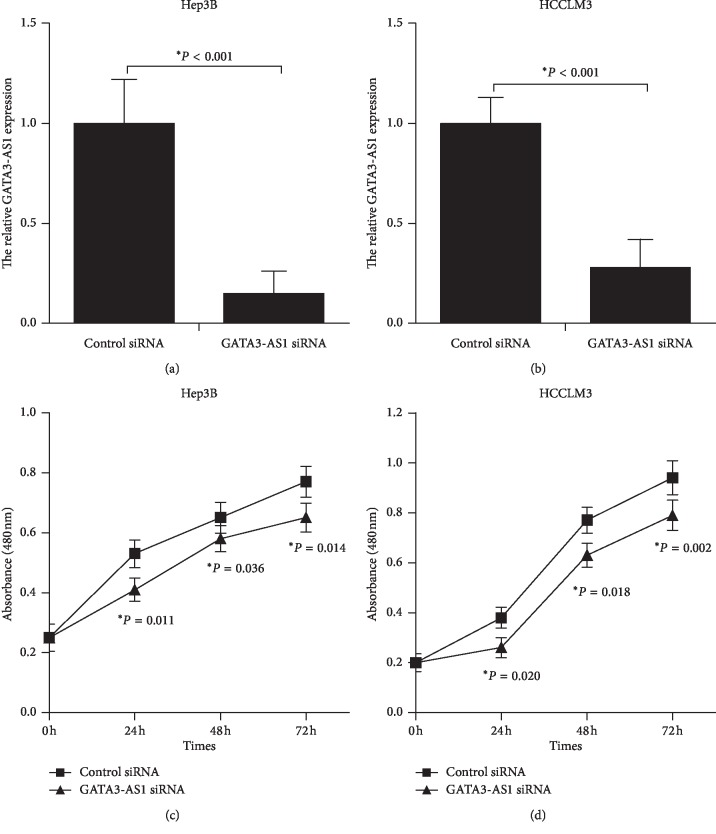
GATA3-AS1 knockdown inhibits cell proliferation in Hep3B and HCCLM3 cells. (a, b) Hep3B and HCCLM3 cells were transfected with GATA3-AS1 siRNA, and control siRNA was used as a negative control. GATA3-AS1 expression levels were analyzed after 48 h of transfection by RT-qPCR. (c) The CCK-8 assay was performed to assess the growth ability of Hep3B cells after treated with GATA3-AS1 siRNA/control siRNA. (d) GATA3-AS1 knockdown could inhibit cell proliferation in HCCLM3 cells. CCK-8: cell counting kit-8, siRNA: small interfering RNA. The results were showed as the mean ± SD from at least three independent experiments; ^*∗*^*P* < 0.05.

**Figure 4:**
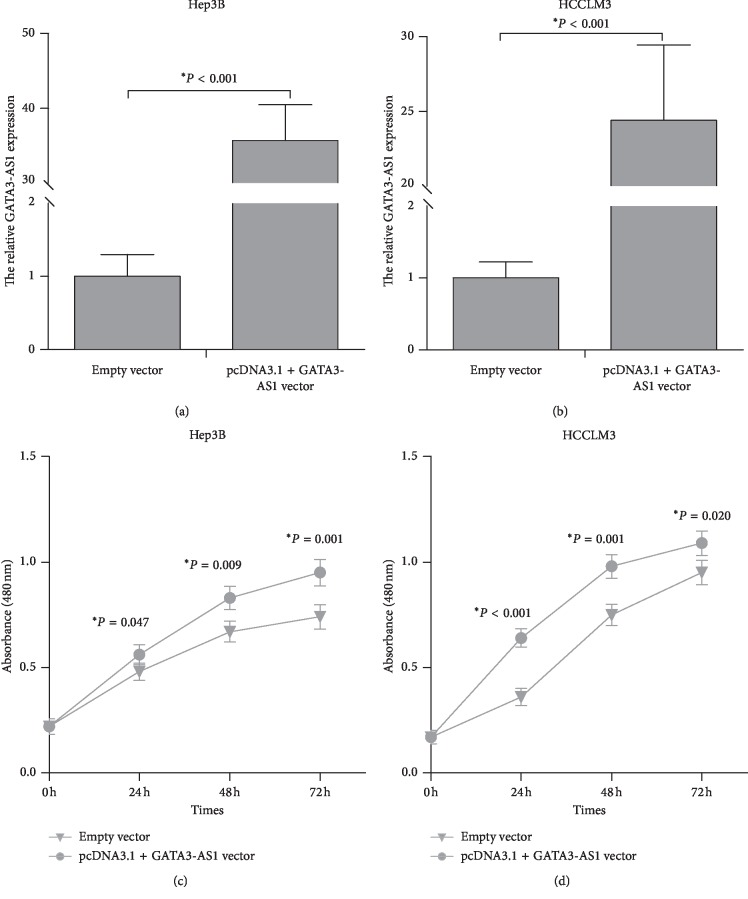
GATA3-AS1 overexpression promotes cell proliferation in Hep3B and HCCLM3 cells. (a, b) pcDNA3.1 + GATA3-AS1 vector markedly increased GATA3-AS1 expression in Hep3B and HCCLM3 cell lines. (c) The CCK-8 assay was performed to evaluate the growth ability of Hep3B cells after transfection of pcDNA3.1 + GATA3-AS1 vector/empty vector. (d) GATA3-AS1 overexpression promoted cell proliferation in HCCLM3 cells. The results were showed as the mean ± SD from at least three independent experiments; ^*∗*^*P* < 0.05.

To examine the biological function of GATA3-AS1 in regulating HCC cell proliferation, Hep3B and HCCLM3 cells transfected with GATA3-AS1 siRNA/control siRNA or pcDNA3.1 + GATA3-AS1 vector/empty vector were analyzed using CCK-8 assay. GATA3-AS1 siRNA significantly decreased GATA3-AS1 expression levels in Hep3B and HCCLM3 cell lines (Figures 3(a) and 3(b), *P* < 0.05). GATA3-AS1 knockdown obviously inhibited cell proliferation in Hep3B and HCCLM3 cells (Figures 3(c) and 3(d), *P* < 0.05). Oppositely, pcDNA3.1 + GATA3-AS1 vector markedly increased GATA3-AS1 expression in Hep3B and HCCLM3 cell lines (Figures 4(a) and 4(b), *P* < 0.05). GATA3-AS1 overexpression notably promoted cell proliferation in Hep3B and HCCLM3 cells (Figures 4(c) and 4(d), *P* < 0.05). These data demonstrated that GATA3-AS1 contributes to cell proliferation in HCC.

### 3.4. GATA3-AS1 Facilitates Cell Metastasis in Hep3B and HCCLM3 Cells

**Figure 5:**
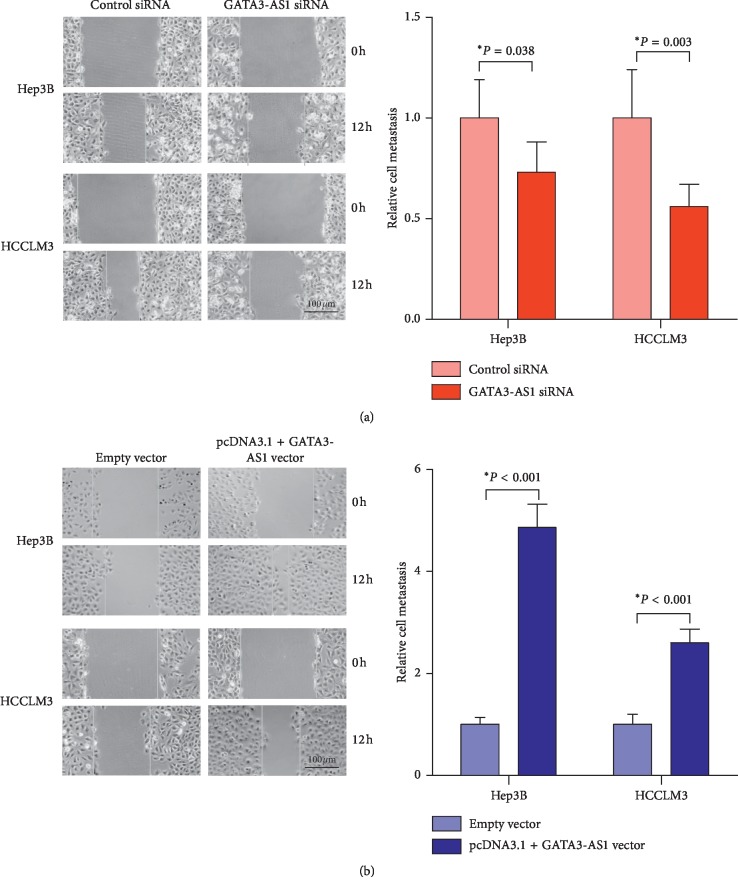
GATA3-AS1 facilitates cell metastasis in Hep3B and HCCLM3 cells. (a) The wound-healing assay was employed to determine Hep3B and HCCLM3 cell migration after treatment with GATA3-AS1 siRNA/control siRNA. (b) Hep3B and HCCLM3 cells transfected with pcDNA3.1 + GATA3-AS1 vector showed longer distance of cell migration compared with cells transfected with empty vector. Data were expressed as the mean ± SD (*n* = 3). Bar = 100 *μ*m and ^*∗*^*P* < 0.05.

Wound scratch assay was used to evaluate metastatic ability in HCC cells. The distance of cell migration in Hep3B cells transfected with GATA3-AS1 siRNA was significantly shorter compared with cells treated with control siRNA (Figure 5(a), *P* < 0.05). Similar results were found in HCCLM3 cells after transfection of GATA3-AS1 siRNA/control siRNA (Figure 5(a), *P* < 0.05). By contrast, Hep3B cells transfected with pcDNA3.1 + GATA3-AS1 vector showed longer distance of cell migration compared with cells treated with empty vector (Figure 5(b), *P* < 0.05). Similar results were also shown in HCCLM3 cells after transfection of pcDNA3.1 + GATA3-AS1 vector/empty vector (Figure 5(b), *P* < 0.05). These data demonstrated that GATA3-AS1 promotes cell metastasis in HCC.

### 3.5. GATA3-AS1 Suppresses PTEN, CDKN1A, and TP53 Expression in HCC Cells

**Figure 6:**
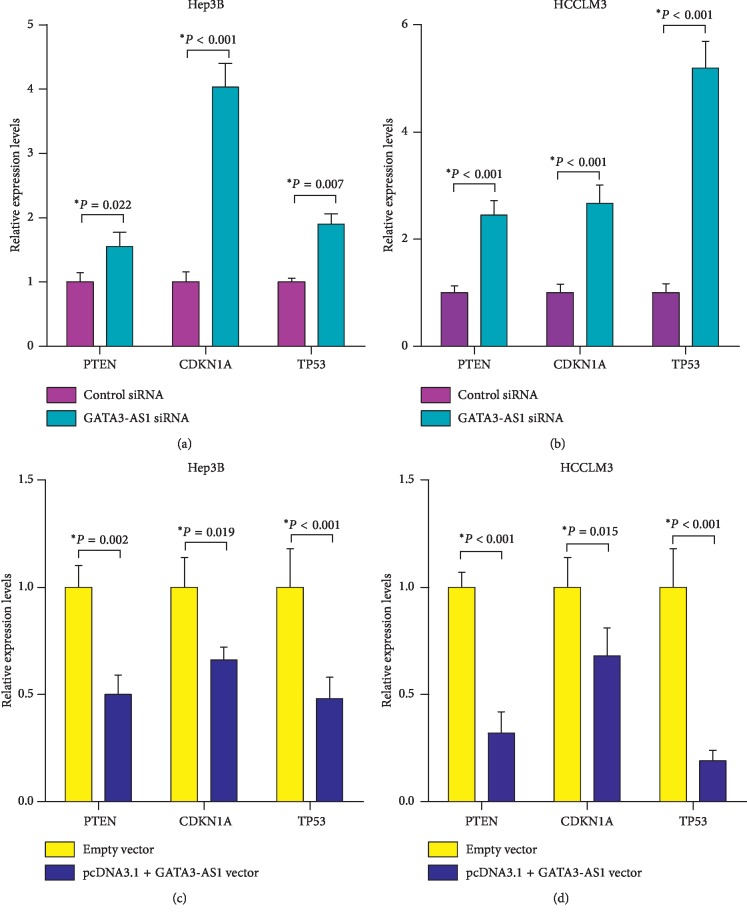
GATA3-AS1 suppresses PTEN, CDKN1A, and TP53 expression in HCC cells. (a) RT-qPCR analysis was used to detect PTEN, CDKN1A, and TP53 expression in Hep3B and HCCLM3 cells after treated with GATA3-AS1 siRNA/control siRNA. Expression of each gene was normalized to that of the ACTB level. (b) Restoration of GATA3-AS1 decreased PTEN, CDKN1A, and TP53 expression. PTEN: phosphatase and tensin homolog, CDKN1A: cyclin-dependent kinase inhibitor 1A, and TP53: tumor protein p53. Each value represented the mean ± SD from at least three independent experiments; ^*∗*^*P* < 0.05.

To investigate the molecular mechanisms underlying the GATA3-AS1-mediated increase in HCC cell proliferation and metastasis, the expression levels of tumor-suppressive genes that play inhibitory roles in HCC progression were evaluated by RT-qPCR, including phosphatase and tensin homolog (PTEN) [21], cyclin-dependent kinase inhibitor 1A (CDKN1A) [22], and tumor protein p53 (TP53) [23]. As shown in Figures 6(a) and 6(b), GATA3-AS1 knockdown resulted in upregulation of PTEN, CDKN1A, and TP53 in Hep3B and HCCLM3 cells (*P* < 0.05), while restoration of GATA3-AS1 notably decreased PTEN, CDKN1A and TP53 expression (Figures 6(c) and 6(d), *P* < 0.05). These data demonstrated that GATA3-AS1 promotes cell proliferation and metastasis in HCC by suppression of PTEN, CDKN1A and TP53.

## 4. Discussion

Recent research studies suggested that mammalian transcriptome is more complex than protein-coding genes, and global transcriptome analysis provides detail evidences that a large proportion of human genome can generate transcripts from both strands [24]. More and more studies recently have demonstrated that lncRNAs participate in the regulation of cellular growth, metabolism, apoptosis, invasion, and metastasis [25]. For example, a liver-enriched lncRNA, liver-specific triglyceride regulator (LSTR), can regulate systemic lipid metabolism in mice [26]. Downregulation of lncRNA maternally expressed 3 (MEG3) has an effect on suppressing trophoblast cell migration and promoting apoptosis [27]. The lncRNA H19 imprinted maternally expressed transcript (H19) is significantly upregulated in hepatocellular carcinoma, and confers growth, migration, and invasion advantages on cancer cells [28]. Evidences have indicated that the antisense lncRNAs are participated in complicate pathophysiological processes of human diseases [29]. Thus, we hypothesized that antisense lncRNAs may play crucial roles in progression of HCC.

GATA3-AS1 is a newly antisense lncRNAs, which first identified from human CD4+ T-cell subsets [30]. Subsequently, Zhu et al. [31] applied microarray to analyze lncRNAs expression profiles in human bladder cancer and matched normal bladder tissues and found GATA3-AS1 is remarkably upregulated in tumor tissues. Recently, Gibbons et al. [18] demonstrated that GASTA3-AS1 significantly contributes to TH2 development. However, the expression pattern and role of GATA3-AS1 are largely unknown in HCC until now. In this study, we identified that the GATA3-AS1 expression is commonly upregulated in HCC tissues and Hep3B and HCCLM3 cells, indicating that GATA3-AS1 might have an important function in the progression of HCC. Besides, the elevated GATA3-AS1 expression was closely correlated with larger tumor size, advanced TNM stage, and more lymph node metastasis, suggesting that upregulation of GATA3-AS1 represented an aggressive phenotype of HCC. Subsequently, *in vitro* loss- and gain-of-function experiments revealed that overexpression of GATA3-AS1 contributed to HCC cell proliferation and metastasis, whereas knockdown of GATA3-AS1 inhibited cell growth and metastasis. These data strongly demonstrated that GATA3-AS1 acts as an oncogenic role in the progression of HCC.

TP53 is known as a tumor suppressor gene in various types of human cancer, and low expression of TP53 is associated with tumor progression and poor prognosis of HCC [32, 33]. PTEN is a plasma membrane lipid phosphatase, which is identified as a tumor suppressor gene in multiple types of neoplasm [34]. In recent years, numerous previous studies have demonstrated that downregulated PTEN expression is associated with poor overall survival and carcinogenesis in HCC. CDKN1A is regarded as a key inhibitor of cell cycle, mediator of DNA damage, and effector of the TP53, displaying an important role in the progression of HCC [35]. To investigate the molecular mechanisms underlying the GATA3-AS1-mediated increase in HCC cell proliferation and metastasis, the expression levels of TP53, PTEN, and CDKN1A were evaluated by RT-qPCR. The results showed that GATA3-AS1 inhibition significantly elevated PTEN, CDKN1A, and TP53 expression levels in HCC cells, while restoration of GATA3-AS1 decreased PTEN, CDKN1A, and TP53 expression. All of these data sufficiently demonstrated that GATA3-AS1 promotes cell proliferation and metastasis in HCC by suppression of PTEN, CDKN1A, and TP53. Some limitations of the study have to be improved in the further research. First, the HCC specimens used in our study are small. Second, how GATA3-AS1 regulates PTEN, CDKN1A, and TP53 remains unclear. Moreover, it is essential to study the role of GATA3-AS1 in more other gastrointestinal cancers in further work, such as gastric cancer, pancreatic cancer, gallbladder cancer, and colorectal cancer.

In conclusion, our results suggested that GATA3-AS1 promotes cell proliferation and metastasis in HCC by suppression of PTEN, CDKN1A, and TP53. Our findings contribute to a better understanding of the importance of GATA3-AS1 in HCC progression and provide a promising lncRNA-based targeted approach for HCC treatment.

## Figures and Tables

**Table 1:** The correlations between lncRNA GATA3-AS1 expression and clinicopathologic variables in patients with hepatocellular carcinoma.

Variables	Cases	GATA3-AS1		*χ* ^2^	*P*
		Low	High		
Gender					
Male	51	19	32	0.442	0.506
Female	29	13	16		

Age (years)					
<55	35	17	18	1.905	0.168
≥55	45	15	30		

Tumor size (cm)					
<5	26	16	10	7.445	0.006^*∗*^
≥5	54	16	38		

Differentiation					
Low	46	17	29	1.838	0.372
Moderate	29	14	15		
High	5	1	4		

Liver cirrhosis					
No	23	9	14	0.010	0.920
Yes	57	23	34		

Lymph node metastasis					
No	41	25	16	14.626	<0.001^*∗*^
Yes	39	7	32		

Venous invasion					
No	18	10	8	2.342	0.126
Yes	62	22	40		

TNM stage					
I + II	27	17	10	8.954	0.003^*∗*^
III + IV	53	15	38		

LncRNA: long noncoding RNA, GATA3-AS1: GATA3 antisense RNA 1. ^*∗*^*P* < 0.05.

**Table 2:** The specific primer sequences for RT-qPCR.

Gene	Sense (5′-3′)	Antisense (5′-3′)
GATA3-AS1	TTGTTCCCTCTTCGCTCCT	TTGTTCCTTCACCGCATG
PTEN	ACCATAACCCACCACAGC	CAGTTCGTCCCTTTCCAG
CDKN1A	TCTACATCTTCTGCCTTAG	AAATGCCCAGCACTCTTA
TP53	TAAGGGTTAGTTTACAATC	TGCCAGCATTTCACAGAT
ACTB	ACTTAGTTGCGTTACACC	GTCACCTTCACCGTTCCA

RT-qPCR: real-time quantitative PCR, PTEN: phosphatase and tensin homolog, CDKN1A: cyclin-dependent kinase inhibitor 1A, TP53: tumor protein p53, and ACTB: actin beta.

## Data Availability

The data used to support the findings of this study are available from the corresponding author upon request.
